# Assessment of hydrogeochemistry in groundwater using water quality index model and indices approaches

**DOI:** 10.1016/j.heliyon.2023.e19668

**Published:** 2023-09-09

**Authors:** Md Galal Uddin, Mir Talas Mahammad Diganta, Abdul Majed Sajib, Md. Abu Hasan, Md. Moniruzzaman, Azizur Rahman, Agnieszka I. Olbert, Md Moniruzzaman

**Affiliations:** a Civil Engineering, School of Engineering, College of Science and Engineering, University of Galway, Ireland; b Ryan Institute, University of Galway, Ireland; c MaREI Research Centre, University of Galway, Ireland; d Eco-HydroInformatics Research Group (EHIRG), Civil Engineering, University of Galway, Ireland; e Department of Geography and Environment, Jagannath University, Dhaka, Bangladesh; f Bangladesh Reference Institution for Chemical Measurements (BRiCM), Dr. Qudrat-e- Khuda Road, Dhanmondi, Dhaka 1205, Bangladesh; g School of Computing, Mathematics and Engineering, Charles Sturt University, Wagga Wagga, Australia; h The Gulbali Institute of Agriculture, Water and Environment, Charles Sturt University, Wagga Wagga, Australia

**Keywords:** Groundwater quality, Hydrogeochemistry, Water quality index, CCME index, Irrigation indices, Nuclear power plant

## Abstract

Groundwater resources around the world required periodic monitoring in order to ensure the safe and sustainable utilization for humans by keeping the good status of water quality. However, this could be a daunting task for developing countries due to the insufficient data in spatiotemporal resolution. Therefore, this research work aimed to assess groundwater quality in terms of drinking and irrigation purposes at the adjacent part of the Rooppur Nuclear Power Plant (RNPP) in Bangladesh. For the purposes of achieving the aim of this study, nine groundwater samples were collected seasonally (dry and wet season) and seventeen hydro-geochemical indicators were analyzed, including Temperature (Temp.), pH, electrical conductivity (EC), total dissolved solids (TDS), total alkalinity (TA), total hardness (TH), total organic carbon (TOC), bicarbonate (HCO_3_^−^), chloride (Cl^−^), phosphate (PO_4_^3−^), sulfate (SO_4_^2−^), nitrite (NO_2_^−^), nitrate (NO_3_^−^), sodium (Na^+^), potassium (K^+^), calcium (Ca^2+^) and magnesium (Mg^2+^). The present study utilized the Canadian Council of Ministers of the Environment water quality index (CCME-WQI) model to assess water quality for drinking purposes. In addition, nine indices including EC, TDS, TH, sodium adsorption ratio (SAR), percent sodium (Na%), permeability index (PI), Kelley's ratio (KR), magnesium hazard ratio (MHR), soluble sodium percentage (SSP), and Residual sodium carbonate (RSC) were used in this research for assessing the water quality for irrigation purposes. The computed mean CCME-WQI score found higher during the dry season (ranges 48 to 74) than the wet season (ranges 40 to 65). Moreover, CCME-WQI model ranked groundwater quality between the “poor” and “marginal” categories during the wet season implying unsuitable water for human consumption. Like CCME-WQI model, majority of the irrigation index also demonstrated suitable water for crop cultivation during dry season. The findings of this research indicate that it requires additional care to improve the monitoring programme for protecting groundwater quality in the RNPP area. Insightful information from this study might be useful as baseline for national strategic planners in order to protect groundwater resources during the any emergencies associated with RNPP.

## 1 Introduction

The United Nations sustainable development goal (SDG) no. 6 is one of the fundamental pillars for achieving sustainable future for humanity. The SDG 6 has the ambitious mission to ensure availability and sustainable management of water and sanitation for all by 2030. Therefore, it is crucial to ensure the safe utilization of water resources by maintaining the “good” status of the water quality in both ground and surface water [ [[1], [2], [3], [4], [5]]]. However, the quality of surface water is rapidly declining day-by-day due to the direct discharge of effluents in waterbodies such as canal, rivers, estuaries and bays [ [[6], [7], [8], [9]]]. Therefore, the groundwater resources have been utilizing as a major replenishable source of freshwater for human consumption, farming and industrial application around the world [ [[10], [11], [12], [13]]]. At present, groundwater resources meet the global demand of drinking water by 65% and the need of irrigation water by 43% [14].

Nevertheless, recent studies have reported that the world's groundwater level has declined significantly over the decades due to over-abstraction for various purposes such as drinking, industrial, and agricultural activities [15]. Moreover, the recent UN World Water Development Report have stated that groundwater quality around the world especially in developing countries are facing tremendous challenges from intensified application of agrochemicals, rapid urbanization, improper disposal of industrial waste, uncontrolled leaching of leachate from landfill sites and climate change induced saltwater intrusion [16]. Therefore, considering the present status of groundwater quality, it is required to have a delicate periodic monitoring practise to keep the “good” status. It is also worth mentioning that the safeguarding of groundwater quality is also the key element in achieving SDG 2 (zero hunger) and SDG 3 (good health and well-being) as well.

For the purposes of groundwater quality monitoring, it is required to assess the hydrochemistry and its quality periodically. Typically, the hydrochemistry of groundwater is influenced by a number of natural factors (e.g., climate, hydrogeology, geology, hydrodynamic conditions, and precipitation etc.), and anthropogenic factors (e.g. industrial effluents, pesticides, and fertilizers from agriculture production within the aquifer area) [17,18]. Moreover, the hydro-geochemical properties of groundwater are the major controlling factors for groundwater quality [19,20]. In this regard, most straightforward approach is to compare the groundwater quality indicators to their guideline levels [21,22]. Recently, for the purposes of monitoring groundwater quality, a series of tools and techniques have been utilized in groundwater research. The water quality index (WQI) is one of the most widely used techniques for assessing the suitability of groundwater quality [ [[23], [24], [25], [26]]]. It is one of the most convenient and trending method, which convert a vast water quality information into a numeric score, reflecting the suitability of water resources for both drinking and irrigation purposes [ [[27], [28], [29], [30], [31], [32]]]. To date, several WQI models have been developed for assessing water quality (e.g. groundwater, surface water i.e. lake, river, coastal etc.) [8,33]. Details of the WQIs models and their applications discussed in Uddin et al. (2021). In addition to WQI models, several studies have employed multivariate statistical analysis (e.g., Principal Component Analysis-PCA, Cluster Analysis-CA) for identifying contamination sources and the pattern for homogeneous water quality indicators [ [[34], [35], [36], [37], [38], [39], [40]]]. Like other countries around the world, the groundwater resources in Bangladesh meet the demand of approximately 90% of drinking water and 75% of irrigation water [41]. Due to its subtropical monsoon climate, relatively shallow water table (depth of 1–10 m below the surface), aquifer storage capacity, consumption rate, and changes in volume and distribution of groundwater recharge conditions, groundwater is easily accessible throughout the year for public use [42]. Accoreding to literature, approximately 32 km^3^/year water is directly cast-off from groundwater resources of Bangladesh that is equivalent to around 4% of the global withdrawal of groundwater [43]. However, several studies have stated that the excessive abstraction of groundwater resources from the semi-confined and quaternary-alluvial/deltaic aquifers have created a water stress situation [44]. Furthermore, with the recent rapid urbanization and industrialization in Bangladesh, the groundwater quality is degrading drastically [19,45]. Therefore, groundwater resources of Bangladesh are in desperate need of frequent monitoring in order to keep the quality of water useable for human.

Although recently there has been an increasing trend of water quality monitoring in Bangladesh especially for groundwater quality, that was not the case a few years back. Consequently, there is a significant data gap for majority of the regions in Bangladesh and some of these regions are regarded as extreme hotspot in terms of their immense ecological and economical significance. Among these regions, RNPP area is one of the crucial one, which will be the first nuclear plant in Bangladesh to support the national grid system for meeting the nationwide electricity demand [46]. The construction of the RNPP began during the year 2017 and at present, the power plant is planning to begin its operation within 2024 [47]. Therefore, this area has been a region of interest for numerous scientific research and several studies have so far reported the status of groundwater [ [4,5,[48], [49], [50]]]. However, all these studies have reported the status of groundwater quality around the RNPP area from the beginning of the construction phase [4,5] to this date [ [[48], [49], [50]]]. The earlier study regarding the groundwater quality in this region can be traced to Ref. [51]. However, there is no information available on the groundwater quality between the year of 2011–2016. This conspicuously indicated a literature and data gap on the quality of groundwater resources prior to the RNPP construction project. Therefore, it is required to generate a baseline data on the groundwater quality that would assist policy makers for tracking any change within the groundwater resources over the period and to initiate necessary actions during any catastrophic events arising from RNPP area.

Considering the above-mentioned issues, the main aim of the research was to assess the suitability of groundwater in terms of drinking and irrigation purposes in the adjacent area of the RNPP during the pre RNPP phase (2014–2015). Additionally, this study considered the seasonality issue of Bangladesh for appraising the groundwater quality, which remained out of sight by the previous literature. Typically, the seasonality of Bangladesh is divided into dry (November to March) and wet (April to September) season where the groundwater recharge is higher during wet season than the dry season due to the monsoon-induced heavy rainfall [52]. Therefore, in order to assess the impact of seasonality in the groundwater's suitability, following two hypothesis were formulated: (i) *null hypothesis (H*_*0*_*)*: the suitability of groundwater would vary due to seasonality and (ii) *alternate hypothesis (H*_*a*_*)*: despite seasonality, the suitability of groundwater would remain unchanged. Finally, a number of objectives were carried out in order to achieve the goal of this study, which are as follows.

(i) to assess groundwater suitability in terms of drinking and irrigation purposes using various indices approaches,
(ii) to determine the hydrogeochemical characteristics of groundwater in the adjacent aquifers of the RNPP area utilizing multivariate statistical analysis, and
(iii) to analyse the spatio-temporal variability of groundwater hydrochemistry using state-of-the-art GIS technique.

The paper is composed of four sections. The first section provides a brief overview of the research background, rationality, and aims and objectives. Section two describes the details of the tools and techniques that are used in this study. The third section contains the results of this study and discussion in detail. The fourth section provides a summary of the findings and recommendations.

## 2 Materials and methods

### 2.1 Study area and hydrogeology

**Fig. 1:**
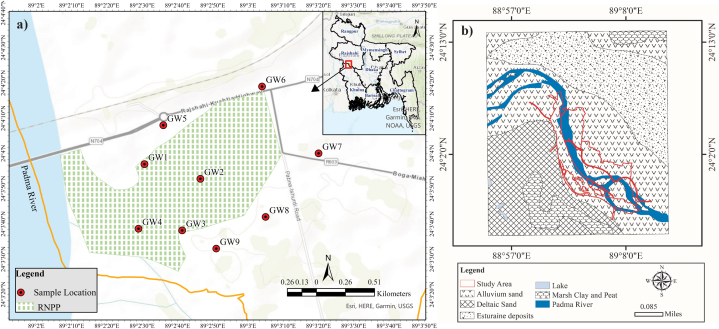
a) Location of Ruppar nuclear power plant and sampling locations; b) Geological characteristics of the aquifers at the sampling location.

The research site is a semi-urban area, situated within the geographic coordinates of 24°03′ N to 24°15′ N latitude and 89°00′ E to 89°11′ E longitude, encompassing a total land area of approximately 246.90 km^2^ (Fig. 1a). The sample locations are located in the Ishwardi sub-district (upazila) of Pabna district under Rajshahi Division of Bangladesh. Geographically, the western boundary of the study domain is adjacent to the Padma River. Following the classification scheme of Koppen, the study domain has a humid sub-tropical climate that is characterized by mild winter during the dry season and the wet season has a warmer summer followed by heavy monsoon [51]. The surface deposit of area is classified as the Ganges River floodplain that are accumulated through the fluvial processes mostly from Ganges and Jamuna River system [53,54]. However, alluvial sands and estuarine deposits are the primary characterization of the study area (Fig. 1b). Usually, the topography of this region appears to be flat but slightly higher ridges and shallow depression can be found in some places [51]. A number of lowlands within the region collect rainwater from the surrounding catchments and supply water to the Padma River and to streams, beels, canal and ponds around it. These surface waterbodies retain water until any secondary movement such as over land runoff or vertical infiltration towards the subsurface takes place.

In terms of metrological attributes, Fig. S1 provides the statistical description of metrological variables (maximum and minimum air temperature and rainfall) over the study domain. These metrological data was retrieved from Bangladesh Metrological Department (http://live.bmd.gov.bd/). Throughout the study period, the maximum air temperature varied from between 24 °C and 35.8 °C and the minimum air temperature varied between 10 °C and 26 °C (Fig. S1a). In terms of rainfall, this region received an annual total rainfall of 1656.2 mm whereas the highest rainfall recorded during the wet season (July: 335.6 mm) and lowest during the dry season (January: 8.1 mm) (Fig. S1b). According to literature, the rate of evaporation usually ranges from 18 to 140.2 mm in the studied region [51]. In the case of the aquifer, the depth of the water table varies between 1.99 and 9.95 m in the study domain [55]. Typically, the groundwater table rises from 3 to 6 m from May to July due to heavy rainfall followed by a stable decrease from August to October, which is attributed to the rejection of recharge as a result of the aquifer reaching its capacity [5] (Fig. S1b). Additionally, previous studies have reported that the groundwater flows from the northwestern corner of the aquifer towards to towards the southeastern corner [56]. According to Ref. [4], groundwater is the primary source of water supply for domestic, agricultural, and industrial purposes in this area. Approximately 93.6% of general household activities are being full filed by lifting drinking water from the wells while the remaining 6.4% from obtained from alternative sources [5].

### 2.2 Groundwater sampling and analysis

For the purposes of collecting groundwater samples, this study followed the protocols outlined in “Environmental Conservation Act 1995″ of Bangladesh that includes various essential components. These components encompass sampling, sampling locations (spatial ranges) and frequency, water quality indicators, analytical procedures, quality control, and data management. Each component is detailed within the regulations that provides a comprehensive guideline for water sampling. By adhering to these national regulations, present research ensures a standardized approach for selecting the sampling sites. The specific requirements and procedures for each component can be referenced in “Environmental Conservation Act 1995" [57].

In order to assess the drinking and irrigation water quality status, groundwater samples were collected for both dry and wet season during 2014–2015 in triplicate from each of the nine shallow, deep boreholes and hand-dug wells equipped with a pump by maintaining standard guidelines of [58]. The geographic coordinates of each groundwater well were documented using a handheld Garman eTrex10 global positioning system (GPS). These wells were subsequently identified with labels ranging from GW1 to GW9 (Fig. 1a). Out of nine water wells, four (GW1, GW2, GW3, and GW4) were situated within the confines of the Rooppur nuclear power plant (RNPP), while the remaining wells (GW5, GW6, GW7, GW8, and GW9) were located in the adjacent residential area of RNPP (refer to Fig. 1a). Groundwater samples from wells were collected at various depths, such as from 2.5 to 4.25 m (dry season) and 2.75–4.0 m (wet season) below groundwater level (Table 3). Following a 10-min pumping period, samples of groundwater were gathered and subsequently placed in PolyEthylene bottles with a capacity of 500 mL. Prior to this, the bottles were pre-washed in a solution of 10% nitric acid for a period of 24 h and then rinsed with laboratory grade deionized water at a temperature of 10 °C. Additionally, the YSI Professional Plus Digital Multimeter (SKU6050000, YSI Incorporated, USA; accuracy: ±0.2% of field measurement) utilized to take *in-situ* readings of the groundwater's Temp., pH, and EC in each sampling location.

#### 2.2.1 Samples storage and preparation

Following the collection of the samples, they were immediately transported to the laboratory and held at a temperature of 4 °C for further examination. Table 1 provides a summary of all the studied indicators, along with the respective procedures and instruments that utilized for conducting chemical analysis. Since errors of less than 5% are allowed in analytical procedures [58], an ionic mass balance was used to verify accuracy. All chemical analyses conducted at the Bangladesh Reference Institute for Chemical Measurements (BRiCM; an ISO 9001:2015 accredited national metrology laboratory), which is affiliated with the Bangladesh council of scientific and industrial research (BCSIR).

**Table 1:** Water quality indicators, their units and analytical methods.

Water quality indicators	Units	Adopted analytical method	Analytical instruments/techniques
Temperature (Temp.)	°C	Method 2550 B [58]	YSI Professional plus digital multimeter (Sensor model: ISE-605103)
pH	–	Method 4500-H^+^ B [58]	
Electrical conductivity (EC)	μS/cm	Method 2510 B [58]	YSI Professional plus digital multimeter (Sensor model ISE 6052030)
Total Dissolved solids (TDS)	mg/L	Method 2540 C [58]	Gravimetric method
Total Alkalinity (TA)	mg/L as CaCO3	Method 2320 B [58]	Titrimetric method
Total Hardness (TH)	mg/L as CaCO3	Method 2340 C [58]	EDTA Titrimetric Method
Total Organic Carbon (TOC)	mg/L	Method 5310 B [58]	TOC analyzer (Model no.: Shimadzu TOC-5000 A, Japan)
Bicarbonate (HCO_3_^−^)	mg/L as CaCO3	Method 4500-CO_2_ D [58]	Carbon Dioxide and Forms of Alkalinity by Calculation
Chloride (Cl^−^), Nitrite (NO_2_^−^), Nitrate (NO_3_^−^), Phosphate (PO_4_^3−^), Sulfate (SO_4_^2−^)	mg/L	Method 4110 B [58]	Ion chromatography (IC) (Model no.: Shimadzu 7900 series, Japan)
Sodium (Na^+^), Potassium (K^+^)	mg/L	Method 4500-Cl- G [58]	UV-VIS spectrophotometer with a diode array detector (DAD) (190–1100 nm) (Model no.: Shimadzu 1700, Japan)
Magnesium (Mg^2+^), Calcium (Ca^2+^)	mg/L	Method 3030 A [58]	Atomic Absorption Spectrophotometer (AAS) (Model no.: Shimadzu AA-7000, Japan)

#### 2.2.2 Analysis of water quality (WQ) indicators

The objectives of this study were accomplished through the analysis of seventeen water quality indicators. The BRiCM has implemented a comprehensive quality control and quality assurance system as part of the national monitoring program for water quality. This system is established to ensure the reliability and accuracy of data generated through this establishment. To analyse water quality and its various indicators, BRiCM has adopted the analytical procedures outlined by the APHA-AWWA-WEF [58]. Detailed information regarding the standard analytical procedures can be found in Ref. [58]. Moreover, BRiCM is specifically designed to uphold the highest standards of precision and accuracy, thereby obtaining dependable and trustworthy water quality data from diverse water bodies and groundwater samples across Bangladesh. Detailed information on the utilized analytical procedures for each of the water quality indicators have been presented in Table 1.

### 2.3 Groundwater suitability analysis

#### 2.3.1 For drinking purpose

A number of WQI models utilized by different researcher/organization/country to assess the suitability of drinking water. The details of the WQI models can be found in Ref. [8]. Among them, the Canadian Council of Ministers of the Environment water quality index model (CCME-WQI) model has been extensively utilized in numerous studies to evaluate the quality of groundwater [ [4,[61], [62], [63], [64], [65]]]. For the determination of the suitability analysis of groundwater in terms of drinking purposes, the present study was utilized the CCME-WQI model in approaching to the [4]. The CCME-WQI comprised of three primary components: (i) scope (F1-which defines the percentage of variables that have values outside the range of desirable levels), (ii) frequency (F2- which is determined by ratio of value outside the desirable levels) and (iii) amplitude (F3- which represents the average deviation of unsuccessful test values from their corresponding reference value) [8]. Details of the model can be found in Ref. [8]. Component F1 and F2 were calculated using equations (1), (2) respectively.

(1) $$F_{1}=\left(\frac{number\;of\;failed\;parameters}{total\;number\;of\;parameters}\right)\times 100$$

(2) $$F_{2}=\left(\frac{number\;of\;failed\;tests}{total\;number\;of\;tests}\right)\times 100$$

The F3 values calculated utilizing following multiple phases. The relative deviation of an unsuccessful test from the objective is termed an excursion and calculated following equations (3a) and (3b):

When *i*th test value didn't exceed the particular objective value:

(3a) $$Excursion_{i}=\left(\frac{{failed\;test\;value}_{i}}{{objectives}_{i}}\right)-1$$

When *i*th test value falled below the objectives (guidelines) value, the excursion was calculated by the following equation (3b):

(3b) $$Excursion_{i}=\left(\frac{{objectives}_{i}}{{failed\;test\;value}_{i}}\right)-1$$

After that, the normalized sum of excursion (*nse*) was calculated by the following equation (4):

(4) $$\text{nse}=\left(\frac{\sum_{i=1}^{n}{excursion}_{i}}{total\;number\;of\;tests}\right)$$

Where, n is the total number of test.

Finally, F3 was calculated utilizing the following function (equation (5)) where nse scales the normalized sum of the excursions from objectives to produce a range between 0 and 100:

(5) $$F_{3}=\left(\frac{nse}{0.01\;nse+0.01}\right)$$

Once all components were obtained, the index value was calculated using equation (6):

(6) $$\text{CCME}-\text{WQI}=\left(\sqrt{\frac{F_{1}^{2}+F_{2}^{2}+F_{3}^{2}}{1.732}}\right)$$

The divisor 1.732 normalizes the resultant values to a range between 0 and 100, where 0 indicates the worst status of water quality and 100 denotes the best excellent status of water quality [66]. Details of the classification schemes of the CCME-WQI model are provided Table S1.

#### 2.3.2 Irrigation indices

The majority of people in the studied area rely on groundwater for their agricultural needs; therefore, it is crucial to understand the region's prospective crop yields, soil productivity, etc. In order to assess the suitability of irrigation water the following indices such as EC, TDS, TH, Sodium adsorption ratio (SAR), Percent sodium (Na%), permeability index (PI), Kelley's ratio (KR), Magnesium hazard ratio (MHR), Soluble sodium percentage (SSP) and Residual sodium carbonate (RSC) were utilized in this research. Table 2 provides a concise overview of these indices.

**Table 2:** Irrigation suitability assessment of groundwater.

Indices	Equation	Objective	Classification	Reference
EC (μS/cm)	**-**	**-**	Excellent (<250) Good (250–750) Permissible (750–2000) Doubtful (2000–3000) Unsuitable (>3000)	[67]
TDS (mg/L)	**-**	**-**	Good (<450) Permissible (450–2000) Unsuitable (>2000)	[68]
TH (mg/L)	**-**	**-**	Soft water (<75.0) Moderate (75.0–150) Hard (150–300) Very hard (>300)	[69]
Sodium adsorption ratio (SAR; meq/L)	$SAR=\frac{{Na}^{+}}{\sqrt{\frac{{(Ca}^{2+}+{Mg}^{2+})}{2}}}$	For measuring the alkali threat in irrigation.	Excellent quality (<10.0)	[70]
			Good quality (10.0–18.0)	
			Acceptable quality (18.0–26.0)	
			Unacceptable quality (>26.0)	
Percent sodium (Na%)	$Na\%=\left(\frac{{Na}^{+}+K^{+}}{{Ca}^{2+}+{Mg}^{+}+{Na}^{+}+K^{+}}\right)\times 100$	For evaluating the effect of Na concentration on soil structure and permeability.	Suitable (<30.0%)	[71]
			Marginal suitable (30.0–60.0%)	
			Unsuitable (>60.0%)	
Permeability index (PI)	$PI=\left(\frac{{Na}^{+}+\sqrt{{HCO}_{3}^{-}}}{{Ca}^{2+}+{Mg}^{2+}+{Na}^{+}}\right)\times 100$	For measuring the groundwater suitability for irrigation purposes.	Unsuitable (<25.0)	[72]
			Good (25.0–75.0)	
			Suitable (>75.0)	
Kelley's ratio (KR; meq/L)	$KR=\frac{{Na}^{+}}{{Ca}^{2+}+{Mg}^{2+}}$	For assessing the Na hazard on water quality.	Unsuitable (>1.00)	[73]
			Suitable (<1.00)	
Magnesium hazard ratio (MHR; meq/L)	$MHR=\left(\frac{{Mg}^{2+}\times 100}{{Ca}^{2+}+{Mg}^{2+}}\right)$	For ascertaining the feasible threat from Mg in agriculture.	Suitable (<50.0)	[74]
			Unsuitable (>50.0)	
Soluble sodium percentage (SSP)	$SSP=\left(\frac{{Na}^{+}}{{Ca}^{2+}+{Mg}^{2+}+{Na}^{+}}\right)\times 100$	For determining Na solubility proportion relative to other ions.	Good (<50.0) Unsuitable (>50.0)	[75]
Residual sodium carbonate (RSC; meq/L)	$RSC=\left({CO}_{3}^{2-}+{HCO}_{3}^{-}\right)-\left({Ca}^{2+}+{Mg}^{2+}\right)$	For evaluating the impact of HCO_3_^−^ and CO_3_^2−^ on the groundwater quality due to irrigation.	Suitable (<1.25) Marginal (1.25–2.50) Unsuitable (>2.50)	[76]

**Table 3:** Average concentration of various water quality indicators in groundwater.

	GW1		GW2		GW3		GW4		GW5		GW6		GW7		GW8		GW9		a^b^	b^c^
DGL^a^	2.50	2.75	3.23	3.66	3.00	3.35	2.85	3.05	3.66	3.66	4.20	3.66	3.75	4.00	4.25	3.35	2.95	3.10	–	–
Para.	Dry	Wet	Dry	Wet	Dry	Wet	Dry	Wet	Dry	Wet	Dry	Wet	Dry	Wet	Dry	Wet	Dry	Wet		
Temp.	25.0	29.2	23.0	29.0	17.0	28.4	19.3	28.3	21.0	28.1	22.0	29.3	20.0	30.4	18.6	30.4	19.8	30.5	–	20.0–30.0
pH	8.27	7.66	7.35	7.86	7.05	7.94	7.60	7.98	7.65	7.85	7.28	7.86	8.11	7.87	7.20	7.79	6.90	7.77	6.50–8.50	6.50–8.50
EC	800	876	980	1275	941	645	613	790	678	722	420	730	680	756	417	397	521	554	1500	1000
TDS	77.2	521	123	827	46.8	316	59.1	399	57.0	385	85.2	575	27.6	186	18.1	122	114	770	1500	1000
TA	110	125	103	75.0	95.0	50.0	44.0	66.7	167	158.3	187	316.7	148	150.0	235	41.7	198	58.3	100	–
TH	157	299	175	427	165	276	153	316	155	333	170	296	145	329	160	152	147	219	500	500
TOC	2.41	3.30	9.36	12.8	1.15	1.58	1.15	1.58	0.770	1.06	0.81	1.11	1.83	2.50	2.08	2.85	3.02	4.14	–	–
HCO_3_^−^	55.4	61.9	50.3	36.1	46.4	23.2	23.8	31.8	68.0	79.0	98.3	161	76.7	74.7	123	18.9	97.5	27.5	500	–
Cl^−^	8.38	62.1	13.0	97.4	14.1	49.0	17.3	69.8	11.7	56.8	22.6	49.9	21.7	60.5	14.4	47.9	41.3	127	500	250
PO_4_^3−^	2.19	3.61	7.73	12.8	13.32	22.0	4.10	6.76	3.27	5.40	2.34	3.86	11.6	19.2	4.10	6.77	57.8	95.4	–	6.00
SO_4_^2−^	ND^d^	ND^d^	2.26	5.43	1.27	3.05	0.900	2.16	0.020	0.044	0.530	1.27	0.580	1.39	0.150	0.352	54.8	132	250	250
NO_2_^−^	0.180	0.540	0.223	0.670	0.180	0.540	0.257	0.770	0.237	0.710	0.170	0.510	0.397	1.19	0.197	0.590	0.310	0.930	3.00	1.00
NO_3_^−^	17.1	28.3	<1.00	12.7	<1.00	<1.00	2.67	<1.00	1.06	27.5	3.12	5.06	1.20	16.3	1.12	2.57	9.68	23.0	45.0	45.0
Na^+^	25.5	68.7	57.2	154	22.6	60.9	25.8	69.6	16.7	45.1	8.93	24.1	17.7	47.9	16.8	45.3	103	279	200	200
K^+^	32.0	183	26.9	208	12.4	95.5	18.8	145	14.1	87.9	16.0	99.8	15.7	97.9	14.8	92.6	28.4	177	12.0	12.0
Ca^2+^	7.71	28.6	8.45	34.7	6.24	14.1	14.3	18.0	13.3	14.1	10.0	12.9	26.6	15.8	21.2	9.32	15.4	18.2	200	75.0
Mg^2+^	34.9	19.6	35.4	19.9	28.7	16.1	35.5	19.9	34.1	19.2	34.6	19.5	35.3	19.8	16.3	9.14	28.5	16.0	50.0	35.0

a Depth of Groundwater Level (m b.g.l.).

b WHO standard for drinking water [59].

c Bangladesh standard for drinking water [60].

d Not Detected.

### 2.4 Multivariate statistical approaches

The current study employed the multivariate statistical approach to accomplish its main objective. In recent times, several studies have employed these methodologies to evaluate the hydrogeochemical characteristics of groundwater quality [ [19,[77], [78], [79], [80]]]. Descriptive statistics were used to summarize the groundwater data, including mean, median, minimum, and maximum values for the water quality indicators under study. The study conducted an analysis of the Piper and Gibbs diagrams to determine the predominant hydrochemical water quality indicators and natural factors influencing the composition of groundwater formations, respectively. The *t*-test and analysis of variance (ANOVA) technique have been extensively employed in numerous studies to evaluate statistical fluctuations in the concentration of environmental variables [ [39,40,81,82]]. Numerous recent studies have demonstrated the efficacy of utilizing the Welch's *t*-test and ANOVA analysis [83]. The present study utilized the Welch's *t*-test anad One-way ANOVA analysis, following the methods of Ref. [84], to assess the differences in terms of temporal and spatial resolution, correspondingly, for the assessed water quality indicators.

Table 4 presents a condensed overview of the ANOVA test results. Conversely, the one-sample *t*-test was applied in order to compare the mean levels of each water quality indicators with their corresponding maximum threshold limits. Details of the one-sample *t*-test results can be found in Table 5. In this study, the Pearson's correlation analysis was employed for the purposes of the determination of the statistical association among water quality indicators. All statistical analysis were performed using RStudio 2022.02.0 b y implementing R programming language. However, the analysis of hydro-geochemical data frequently entails the consideration of multiple water quality indicators and therefore, it regarded as a multivariate problem that requires multivariate statistical analysis for identifying the origin of source for mineralization [ [79,80,85]]. In this regard, this study utilized the principal component analysis (PCA) and cluster analysis (CA) for identifying the sources of origin of the hydrogeochemical. Detail of these techniques outlined as follows.

(i) Principal component analysis (PCA)

**Table 4:** Variation of water quality indicators between the sampling sites over the study period using One-way ANOVA analysis (Significance level p < 0.05).

WQ indicators	df	F	p-value
Temp.	8	0.081	0.999
pH	8	0.576	0.776
EC	8	4.79	<0.050
TDS	8	0.409	0.889
TA	8	1.57	0.256
TH	8	0.279	0.957
TOC	8	23.4	<0.05
HCO_3_^−^	8	1.57	0.257
Cl^−^	8	0.387	0.902
PO_4_^3-^	8	12.4	<0.050
SO_4_^2-^	8	5.69	<0.050
NO_2_^−^	8	0.355	0.92
NO_3_^−^	8	1.56	0.25
Na^+^	8	2.25	0.124
K^+^	8	0.205	0.982
Ca^2+^	8	0.378	0.907
Mg^2+^	8	0.498	0.83

**Table 5:** One-sample *t*-test analysis between the mean values and maximum permissible limits of water quality indicators^a^ (Significance level p < 0.05).

WQ indicators	df	t	p-value
Temp.	17.0	−4.46	<0.050
pH	17.0	−9.52	<0.050
EC	17.0	−15.1	<0.050
TDS	17.0	−20.1	<0.050
TA	17.0	1.68	0.110
TH	17.0	−13.2	<0.050
HCO_3_^−^	17.0	−48.6	<0.050
Cl^−^	17.0	−59.5	<0.050
PO_4_^3-^	17.0	−41.8	<0.050
SO_4_^2-^	17.0	−31.1	<0.050
NO_2_^−^	17.0	−36.4	<0.050
NO_3_^−^	17.0	−15.6	<0.050
Na^+^	17.0	−9.05	<0.050
K^+^	17.0	4.09	<0.050
Ca^2+^	17.0	−102	<0.050
Mg^2+^	17.0	−12.4	<0.050

a TOC excluded from analysis for having guideline limits.

The current investigation utilized PCA for the extraction of principal components (PCs). It is a widely utilized technique to reduce the dimensionality within the dataset by extracting a small number of latent factors for analysing relationships among the observed variables [41]. In this study, the relationships among principal components (PCs) were justified through the application of varimax rotation, utilizing the Guttman-Kaiser rule. The extraction of PCs was conducted with the criterion that the eigenvalue exceeded 1.0 [31]. The PCA can be defined through the following equation (7):

(7) $$Z=a_{i1}x_{1j}+a_{i2}x_{2j}+\ldots +a_{im}x_{mj}$$

where, Z stands for the component score, *a, i, j,* m and x are the component loading, component number, sample number, total number of variables and the measured values of variables, respectively.

(ii) Cluster analysis (CA)

The present study utilized CA to classify the homogeneous chemical groups with regard to sampling sites. It is widely employed to categorize chemical indicators or water samples into distinct clusters based on their degree of similarity or dissimilarity [77]. In recent time, a number of studies have utilized the hierarchical agglomerative clustering technique to extract the common relationships between a single sample and the complete data set [11,86]. This can be visually represented through a dendrogram. The present study utilized the Ward's method with squared Euclidean distances, which can be defined as equation (8) and equation (9), respectively [87].

(8) $$D_{ij}^{2}=\sum_{k=1}^{n}{\left(Z_{ik}-Z_{jk}\right)}^{2}$$

(9) $$Z_{ik}=X_{ik}-\frac{U_{k}}{\sigma_{k}}$$

where, *D*^*2*^_*ij*_ donates the squared Euclidean distance between object *i* and object *j*, and *Z*_*ik*_ and *Z*_*jk*_ are the normalized values for variable *k* (for k = 1 …. n), which reduces the effects of differences in measurement units and renders the data dimensionless. Here, *X*_*ik*_ is the measured value, *U*_*k*_ is the average value, and *σ*_*k*_ is standard deviation of the variable *k*.

### 2.5 Spatio-temporal distribution analysis

In this study, advanced geostatistical techniques were utilized to analyse the spatio-temporal distribution of hydrochemistry in groundwater quality. The cutting-edge geographical information system (GIS) was employed in accordance with established procedures of Ref. [88]. Details of the procedures can be found in Ref. [89].

## 3 Results and discussion

### 3.1 Descriptive hydrochemistry of groundwater

**Fig. 2:**
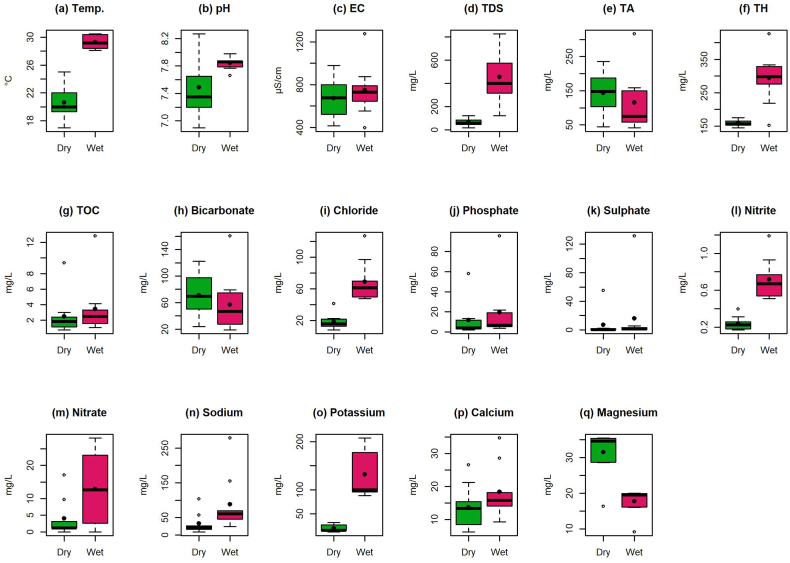
Descriptive statistics for analyzed water quality indicators.

Hydrochemicals serve as valuable indicators for understanding water geochemistry and related regulatory mechanisms, and thus have a diametrical role in the evolution of groundwater quality. Fig. 2 presents the descriptive statistics (minimum, maximum, mean and median) of the water quality indicators. The Whisker boxplot analysis was employed to illustrate the statistical measures.

#### 3.1.1 Physico-chemical assessment of groundwater

In the study area, groundwater Temp. Ranged between 17.0 and 25.0 °C for dry season and 28.1 to 30.5 °C wet season (Fig. 2a; Table 3). The pH values observed in the groundwater samples during the dry and wet seasons were recorded as ranging from 6.90 to 8.27 and 7.66 to 7.98, respectively, as presented in Fig. 2b and Table 3. These results suggest that the groundwater exhibited a slightly alkaline nature, and all samples were found to be within the acceptable range of 6.5–8.5. The slightly alkaline nature indicates that the water could be attributed by the loss of CO_2_, and the rainfall and dissolution of minerals from within the basalt [88]. The average value of EC observed high during the wet season (749 μS/cm) in comparison the dry season (672 μS/cm) (Fig. 2c). Generally, EC values indicate the concentration of dissolved ions that can conduct an electrical current, which changes with temperature and geologically available soluble salts [90].

TDS and TH in sampling station found within the recommended level by Refs. [59,60] (Fig. 2d; Fig. 2f). On the other hand, alkalinity in sampling site GW 1, GW 5, GW 6 and GW 7 exceeded the reference limit of alkalinity (>100 mg/L) in both seasons (Fig. 2e; Table 3). TOC ranged from 0.770 to 12.8 mg/L throughout the study period (Fig. 2g; Table 3). Present finding for Temp., pH, EC, TDS, TA and TH were found consistent with other relevant studies within Bangladesh (Table S2). However, it was not possible to compare the level of TOC as no previous study have reported the TOC level in groundwater. The results of the Welch's *t*-test, as presented in Table S3, indicate that there is a noteworthy statistical variation between seasons for Temp. (df = 10.4, t = 9.98, p < 0.050), TDS (df = 8.35, t = 4.76, p < 0.050) and TH (df = 8.27, t = 5.25, p < 0.050). In addition, the results of the one-way ANOVA showed that there was a significant difference in the concentrations of both EC (df = 8.00, F = 4.79, p < 0.050) and TOC (df = 8, F = 23.4, p < 0.050) between the various sampling locations (Table 4). Lastly, except TA, the average concentration of all remaining water quality indicators exhibited significant variance from their corresponding upper threshold limit (p < 0.050) (Table 5).

#### 3.1.2 Hydrogeo-chemical assessment of groundwater

##### 3.1.2.1 Anions

Based on the results, HCO_3_^−^ is the major anion followed by Cl^−^ > PO_4_^3−^ > SO_4_^2−^ > NO_3_^−^ > NO_2_^−^ in the dry season, while it was found Cl^−^ > HCO_3_^−^ > PO_4_^3−^ > SO_4_^2−^ > NO_3_^−^ > NO_2_^−^ during wet season (Fig. 2h-m). Recently a number of studies have documented similar trend of anion availability in the groundwater of north-west, central-east and Ganges-Padma Basin of Bangladesh (Table S2). The dry season exhibited a range of HCO_3_^−^ ranged from 23.8 to 123 mg/L whereas the wet season demonstrated a variation of HCO_3_^−^ from 18.9 mg/L to 161 mg/L (Fig. 2h). Conversely, the concentration of Cl^−^ varied from 8.38 mg/L to 41.3 mg/L during the dry season and from 47.9 to 127 mg/L during wet season (Fig. 2i; Table 3). According to Ref. [91], Higher Cl^−^ in the aquifer indicates the substantial presence of organic matter, which may produce carbon dioxide, that it is primary source of HCO_3_^−^ in groundwater. Generally, Cl^−^ naturally occurs in groundwater as a result of suspension of salts, soil permeability and porousness, residual food waste, and manures from the farming area [92].

In the case of PO_4_^3−^concentrations, it exceeded reference limit of ECR (6.00 mg/L) across sampling sites in study area throughout the study period while concentration of SO_4_^2−^ varied between 0.020 and 127 mg/L and observed within the reference limit (Fig. 2j; Fig. 2k; Table 3). Additionally, the reported concentration of PO_4_^3−^ in this study was found higher when compared with previous studies (Table S2). The elevated concentration of PO_4_^3−^ might be attributed to the widespread utilization phosphate fertilizer in agricultural practices, leading to the fermentation or decomposition of buried peat deposits and other naturally occurring organic materials [45]. On the other hand, the NO_2_^−^ concentration was varied from 0.180 to 0.397 mg/L with an average of 0.240 mg/L in the dry season, whereas it was found between 0.510 mg/L and 1.19 mg/L with an average of 0.720 mg/L through the wet season (Fig. 2l). Like NO_2_^−^, compared to wet season, higher NO_3_^−^ was found during the wet season, it was varied from 2.57 mg/L to 28.2 mg/L with a mean value of 12.8 mg/L while it was found between 0.090 mg/L and 17.1 mg/L with a mean value of 4.01 mg/L (Fig. 2m). A few studies have revealed similar findings as those of this study [2]. In terms of NO_3_^−^, although higher levels of NO_3_^−^ was found during the wet season than that of during the dry season, it remained within the guideline limit of [59,60]. The higher NO_3_^−^ during the wet season could be due to the infiltration of soil nitrate into the aquifer through various means such as rainfall, streams, and irrigation water [93]. Moreover, findings for NO_3_^−^ are in line with majority of previous studies presented in Table S2. Further statistical tests on the studied anions revealed significant differences in temporal and spatial resolution, such as Cl^−^ (df = 10.1, t = 5.36, p < 0.050) and NO_2_^−^ (df = 9.75, t = 6.11, p < 0.050) with respect to season (Table S3), whereas PO_4_^3−^ (df = 8.00, F = 12.4, p < 0.050) and SO_4_^2−^ (df = 8.00, F = 5.69, p < 0.050) showed statistically significant variation between sampling sites (Table 4). In addition, the mean concentration of all anions under this study demonstrated significant difference with the upper standard threshold limit values (p < 0.050) (Table 5).

##### 3.1.2.2 Cations

In this study, major cations can be hierarchically arranged as follows based on their field measured concentration: Na^+^ > Mg^2+^ > K^+^ > Ca^2+^ during the dry season, and K^+^ > Na^+^ > Ca^2+^ > Mg^2+^ during the wet season (Fig. 2n-q). In contrast to the dry season, the wet season exhibited a prevalence of K^+^ as the dominant ion (Fig. 2o). However, compared to previous literature, present study reported an increased concentration of K^+^ (Table S2). The geological structure of the aquifer might be a contributing factor to the observed phenomenon, as minerals and rocks with relatively high solubility may impart increase levels of K^+^ to the aquifer water. This, in turn, may result in an increase in the concentration of K^+^ in the groundwater [94]. Furthermore, K^+^ exceeded the limit of both standards [59,60] which is recorded of 12 mg/L across all the sampling sites throughout the duration of the study (Table 3). In majority of the sampling sites, Na^+^ concentration was found within permissible limits of WHO (200 mg/L) and ECR (200 mg/L) standards through this study, except for the GW9 (279 mg/L) during the wet season (Fig. 2n; Table 3). It might be a result of Na dissolution from lithogenic minerals and cation exchange between aquifer geochemistry and groundwater [95]. The average concentration of Na^+^ in the groundwater of the study domain is also comparable with the findings of [96,97] (Table S2).

The concentration of Ca^2+^ in groundwater exhibited a range of 6.24 mg/L to 26.6 mg/L with an average value of 13.7 mg/L during the dry season, whereas it was varied between 9.32 mg/L to 34.7 mg/L with an average value of 18.4 mg/L during the wet season (Fig. 2p). Usually, the Ca^2+^ is found in the groundwater as a result of natural dissolution of carbonate rocks such as limestones and dolomites, as well as silicate minerals like plagioclase [98,99]. Regarding Mg^2+^ concentrations in the groundwater, it was found within the prescribed limit of 50.0 mg/L (Fig. 2q; Table 3). Similar to Ca^2+^, it can be enriched in groundwater through the same mineral sources [100]. The present findings for Ca^2+^ and Mg^2+^ are consistent with previous studies by Refs. [4,48,50] for the corresponding region of interest (Table S2). In terms of seasonality of the studied cations, a statistically significant temporal variations observed for K^+^ (df = 8.38, t = 7.07, p < 0.050) and Mg^2+^ (df = 12.6, t = −5.68, p < 0.050) (Table S3). It is noteworthy that there was no significant variation observed in the cations across the sampling sites as indicated in Table 4. However, the mean concentrations for each of the cations exhibited significant deviations from the upper threshold limit values, as presented in Table 5.

### 3.2 Hydrogeochemical facies (HF) and controlling mechanism of groundwater

**Fig. 3:**
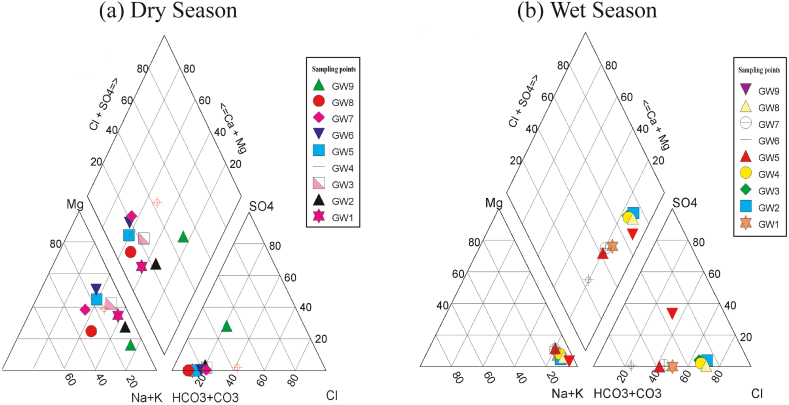
Piper diagram showing the classification of groundwater types in the study area.

The study utilized hydrogeochemical facies (HF) for the purposes of the identification of chemical structure within any groundwater system and to explain the origin and distribution of the primary groundwater categories via a theoretical framework [3]. The current investigation employed Piper's trilinear diagram to determine the dominant cations and anions present in the groundwater within the RNPP area. Fig. 3 displays the outcomes of the HF. The Piper trilinear diagram is a mathematical representation that displays the relative proportions of cationic species (Ca^2+^, Mg^2+^, Na^+^, and K^+^) and anion species (HCO_3_^−^, SO_4_^2−^, and Cl^−^) as a percentage of their total sum. The utilization of trilinear plots presents a highly convenient approach for express the inter-relationship of diverse chemicals within a groundwater system.

As illustrated in Fig. 3a, the cation triangle reveals that approximately 45.0% of samples were categorized as Na^+^-K^+^ whereas 55.0% of samples were identified as cation-free during dry season. In contrast, during the wet season, all samples (100%) were classified as Na^+^-K^+^ dominant (Fig. 3b). During dry season, HCO_3_^−^ type of water found to predominated in all sample of the anion triangle. Notably, a significant variation was observed in the wet season whereas three types of water were assessed, namely Cl^−^ type (56.0%), the HCO_3_^−^ type (33.0%) and the anion free type (11.0%). The prevalence of HCO_3_^−^ and Cl^−^ during the dry and wet respectively could be attributed to their higher loading of HCO_3_^−^ (70.9 mg/L) and Cl^−^ (68.9 mg/L) during those respective seasons. Finally, 66.6% groundwater samples were predominantly influenced by the Ca^2+^-Mg^2+^-HCO_3_^-^ during the dry season indicating dominance of alkaline earth and weak acid which implied the weathering of calcite (CaCO_3_) and dolomite (CaMg(CO_3_)_2_). The findings of the present study on HF are consistent with prior research [50,51]. Based on the results of the HF, the relationship between aquifers geochemistry and groundwater hydrochemistry can be written as follows.

(i) Calcite dissolution: CaCO_3_+H_2_CO_3_=Ca^2+^ + 2HCO_3_^−^
(ii) Dolomite dissolution: (CaMg(CO_3_)_2_ + 2CO_2_ + 2H_2_O=Mg^2+^ + Ca^2+^ + 4HCO_3_^−^

In contrast to seasonal fluctuations, the prevalence of ionic compounds (comprising both cations and anions) during the wet season suggests that the groundwater was rich in alkaline and strong acids, with a majority of samples (56.0%) exhibiting higher levels of strong acids than alkaline earth and weak acids (i.e. Na + K > Ca + Mg; Cl + SO_4_ > CO_3_+ HCO_3_). The results of ionic compounds revealed that the groundwater aquifers were recharged with higher EC (749 μS/cm) and TDS (455 mg/L) values throughout the wet season.

**Fig. 4:**
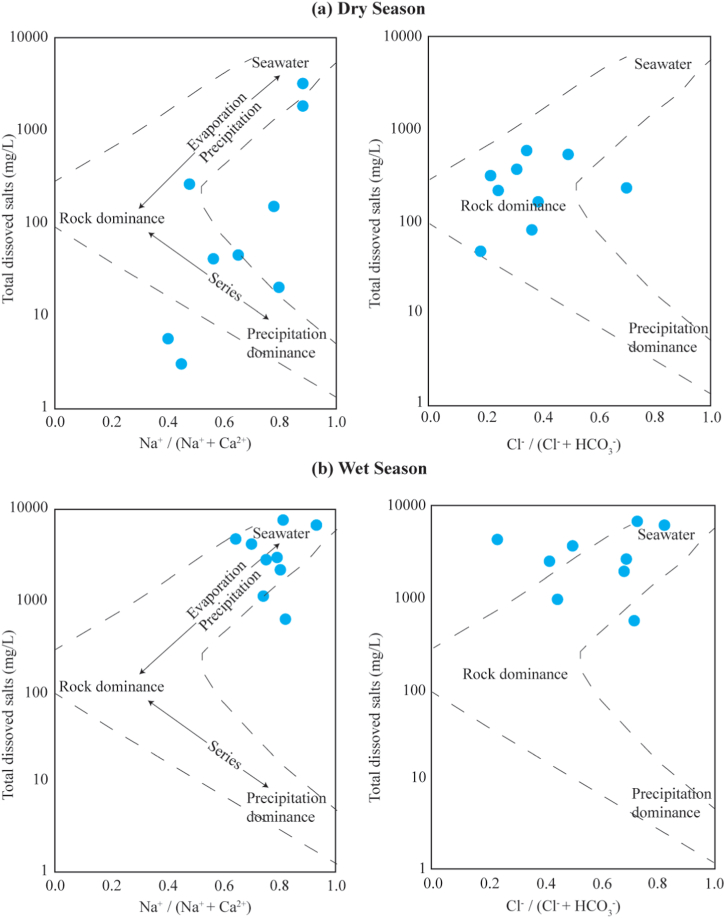
Gibbs plot illustrating the controlling mechanism of the water chemistry for the study area.

Furthermore, the current investigation employed the Gibbs analysis to establish the correlation between the hydro-geochemical composition and the formation process of groundwater [101]. According to the Gibbs analysis, the majority of the sampling sites exhibited rock dominance during the dry season (Fig. 4a). It may be due to the increasing of ions in groundwater from rock-water interaction and mineral dissolution in the investigated area. The data presented in Fig. 4a indicates that two sampling sites (GW2 and GW9) were located within the evaporation zone, whereas three sampling sites (GW3, GW7, and GW8) were situated within the precipitation zone. The Gibbs analysis suggested that the study area may be significantly influenced by various factors such as geological background, precipitation patterns, and anthropogenic activities [102]. In contrast to the dry season, an alternative scenario was examined for the wet season. The findings from the Gibbs analysis indicated that the evaporation process in the studied area was in control of the majority of sampling sites, as illustrated in Fig. 4b. This also pointed out the contribution from the leaching of secondary salts might be due to increasing ion of Na^+^ and Cl^−^ in relation to the increased TDS in the study area from the application of fertilizers [103].

**Fig. 5:**
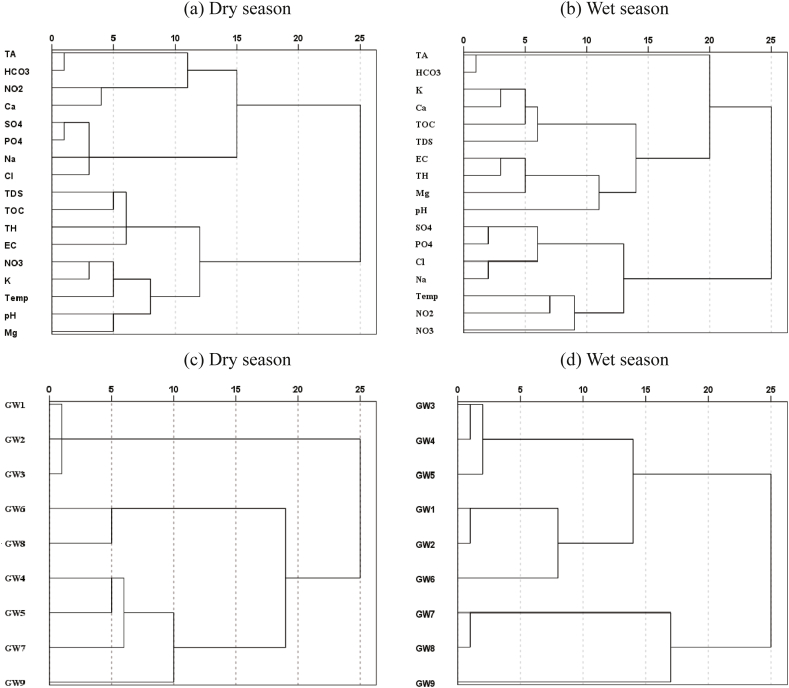
Dendrogram obtained by hierarchical clustering analysis for measured water quality indicators, and sampling sites.

### 3.3 Sources identification of hydrochemical in groundwater

#### 3.3.1 Results of PCs analysis

Principal component analysis (PCA) method was utilized in this study to determine the probabilistic origin of the examined water quality indicators, which extracted five controlling factors with eigenvalues greater than one for the studied datasets in both seasons. Details of the PCA results are provided in Table 6. The loadings of each principal component (PC) were classified into three categories, namely strong, moderate, and weak, according to the loading values of >0.750, 0.750–0.500, and 0.500–0.300 [104]. The present investigation employed PCA with Kaiser normalization to derive five principal components for each season, which were identified as significant factors affecting groundwater in the studied area. The dataset for the dry season was also explainable through five PCs, which explaining 92.8% of the variance. With variables Cl^−^, PO_4_^3−^, SO_4_^2−^, Na^+^ in strong positive loading and TDS in moderate positive loading, the PC1 accounts for 27.3% of total variance. Variables in PC1 were the most critical indicators affecting the groundwater chemistry through anthropogenic and natural occurrences. The potential release of essential indicators into groundwater may occur as a consequence of the interplay between water, soils, and rocks, as well as the weathering of silicate and sodium-bearing minerals [105]. Moreover, the existence of said variables may be attributed to human activities that introduce them into the subterranean water, primarily through the utilization of agrochemicals in the agricultural fields surrounding the examined region [106] The PC2 factor, which solely comprised of two variables (TA and HCO_3_^−^) accounted for 20% contribution towards the overall variance, indicating the occurrence of evaporation, weathering, and rock-water interaction [3]. The PC3, which is the third principal component, accounts for 17.5% of the variance with an eigenvalue of 2.97. The variables included in this component are Temp., pH, NO_3_^−^ and K^+^. The other two PCs (PC4 and PC5) were elucidated for 16.0% and 11.9% of the variance, respectively with strong loading on NO_2_^−^ and Ca^2+^ (PC4) and on TOC and TH (PC5). The origin of NO_2_ and Ca^2+^ might be similar and could be pointed with Calcium Nitrite (Ca(NO_2_)_2_)) which is used to make inorganic Calcium Nitrate (Ca(NO_3_)_2_)), a component in many fertilizer formulations. Moreover, a substantial TH loading is commonly associated with the presence of hardness-inducing ions, such as calcium and magnesium, whereas TOC signifies the dissolution of minerals through both reducing and oxidizing processes [107].

**Table 6:** Varimax rotated factor loadings and communalities of water quality indicators.

WQ Indicators	Dry Season						Wet Season				
	PC1	PC2	PC3	PC4	PC5		PC1	PC2	PC3	PC4	PC5
Temp.	−0.219	−0.068	**0.882**	−0.094	0.351		−0.364	0.326	−0.174	0.565	0.300
pH	−0.581	−0.296	0.572	0.475	−0.117		−0.021	−0.118	−0.012	−0.966	0.201
EC	−0.103	−0.752	−0.008	−0.159	0.398		**0.979**	−0.093	0.104	−0.077	0.001
TDS	0.564	−0.211	0.426	−0.343	0.495		0.629	0.679	0.219	0.063	−0.293
TA	0.134	**0.951**	−0.016	0.078	0.017		−0.038	−0.184	**0.951**	0.066	−0.058
TH	−0.300	−0.008	−0.122	−0.753	0.530		**0.872**	−0.170	0.286	−0.257	0.211
TOC	0.160	−0.156	0.134	−0.076	**0.940**		**0.763**	0.226	−0.355	0.124	−0.088
HCO_3_^−^	0.122	**0.975**	−0.014	0.082	0.016		−0.038	−0.184	**0.951**	0.066	−0.058
Cl^−^	**0.856**	0.266	−0.045	0.264	−0.092		0.369	**0.882**	−0.183	0.122	0.151
PO_4_^3-^	**0.967**	0.116	0.004	0.161	−0.023		−0.175	**0.928**	−0.144	0.112	0.211
SO_4_^2-^	**0.966**	0.185	0.127	0.097	−0.035		−0.160	**0.958**	−0.079	0.179	0.109
NO_2_^−^	0.311	−0.101	−0.079	**0.936**	0.051		−0.015	0.262	−0.048	0.041	**0.956**
NO_3_^−^	0.251	0.000	**0.881**	−0.091	−0.298		0.287	0.199	0.250	0.699	0.264
Na^+^	**0.919**	−0.026	0.164	0.038	0.311		0.224	**0.922**	−0.267	0.149	0.063
K^+^	0.408	−0.153	**0.798**	−0.069	0.307		0.741	0.473	−0.229	0.262	−0.156
Ca^2+^	−0.053	0.425	−0.209	**0.862**	−0.073		**0.939**	0.123	−0.137	0.238	−0.091
Mg^2+^	−0.093	−0.701	0.383	0.159	0.110		0.676	−0.027	0.585	−0.185	0.289
Eigenvalues	4.64	3.40	2.97	2.72	2.01		5.04	4.50	2.71	2.08	1.45
% total variance	27.3	20.0	17.5	16.0	11.9		29.7	26.4	16.0	12.3	8.57
Cumulative % variance	27.3	47.4	64.9	80.9	92.8		29.7	56.1	72.1	84.3	92.9

The first five PCs were accounted for 92.9% of the variance of datasets for the wet season. Details of the PCs are discussed below.

(i) PC1 - explained 29.7% of the total variance with an eigenvalue of 5.04, demonstrated the highest and strong positive loadings of the factors mainly due to EC, TH, TOC, and Ca^2+^; and moderate positive loadings of TDS, K^+^ and Mg^2+^. Inclusion of these indicators in PC1 usually indicate both mineral weathering process and agricultural activities as contributing processes [100].
(ii) PC2 - was dominated by strong positive loadings of Cl^−^, PO_4_^3−^, SO_4_^2−^ and Na^+^ and moderate positive loading of TDS and it elucidated 26.4% of total variance. The higher loadings of Cl^−^, PO_4_^3−^, SO_4_^2−^ and Na^+^ are suggestive of rainfall-induced runoff and infiltration from agricultural lands, primarily due to use of chemical fertilizer, livestock waste and industrial influents [35].
(iii) PC3 - has a total variance of 16.0% with higher positive loading for TA and HCO_3_^−^ and moderate loading for Mg^2+^. Contribution of these variables could be attributed from the substantial amounts of dissolved carbon dioxide and from the water–rock interactions occurring more often between the groundwater and aquifer which results in strong carbonate dissolution [108].
(iv) PC4 and PC5 - accounted for 12.3% and 8.57% of total variance, respectively with moderate positive loadings of Temp. And NO_3_^−^ (PC4) and strong positive loading of NO_2_^−^ (PC5), highlighting human impact on groundwater chemistry through leaching of domestic waste and fertilizers into groundwater [95].

#### 3.3.2 Results of CA analysis

The present study was applied the CA technique to identify the similar water quality sources in study area. Dendrogram in Fig. 5 presents details of the CA results. Regarding the similarity of water quality measures during the dry season, five clusters were identified (Fig. 5a). The Cluster 1 is comprised of TA and HCO_3_^−^, the similar results were found for the PCA analysis during dry season (Table 6). The results of both analysis (CA and PCA) suggested that these two-indicators had homogeneous origin. The Cluster 2 included only Ca^2+^ and NO_2_^−^ indicators. The Cluster 3 comprised of l^−^, PO_4_^3−^, SO_4_^2−^ and Na^+^, which were also identified by PC2 prior to cluster analysis, thus supporting the hypothesis that these water quality indicators might have originated from anthropogenic sources. The group of Cluster 4 suggested that the EC, TDS, TH and TOC are originated from the similar source like natural and anthropogenic sources. The Cluster 5 included NO_3_^−^, K^+^, Temp., pH and Mg^2+^, signifying analogous association in the groundwater.

Dataset of wet season also marked out 5 Clusters in which Cluster 1 (Fig. 5b) containing the elements from PC2 of wet season dataset for PCA (Table 6) backing the statement for PC2. Cluster 2 contained K^+^, Ca^+^, TOC and TDS while Cluster 3 explained the association of EC, TH, pH and Mg^2+^. Cluster 4 pulled the similar variables of PC1 (Cl^−^, PO_4_^3−^, SO_4_^2−^ and Na^+^) which were observed in elevated concentrations further aligning with runoff induced enrichment. Temp., NO_3_^−^ and NO_2_^−^ were grouped in Cluster 5 explaining seasonal difference and associated increment of chemicals used in agriculture through precipitation.

In terms of the similarity of the sampling sites, two significant clusters were found in this study (Fig. 5c; Fig. 5d). During the dry season, cluster 1 composed including sampling sites of GW1, GW2 and GW3 grounded while cluster 2 constructed with sampling sites of GW4, GW5, GW6, GW7, GW8 and GW9 (Fig. 5c). Cluster results indicates to the association of uniform characteristics of grouped sampling sites. Compared to dry season, a significant variation was found in clustering during the wet season. Like dry season, two clusters were identified whereas cluster 1 including most sampling sites except for GW7, GW8 and GW9 (Fig. 5d). Cluster 2 consists of those sampling sites.

#### 3.3.3 Correlation analysis

**Fig. 6:**
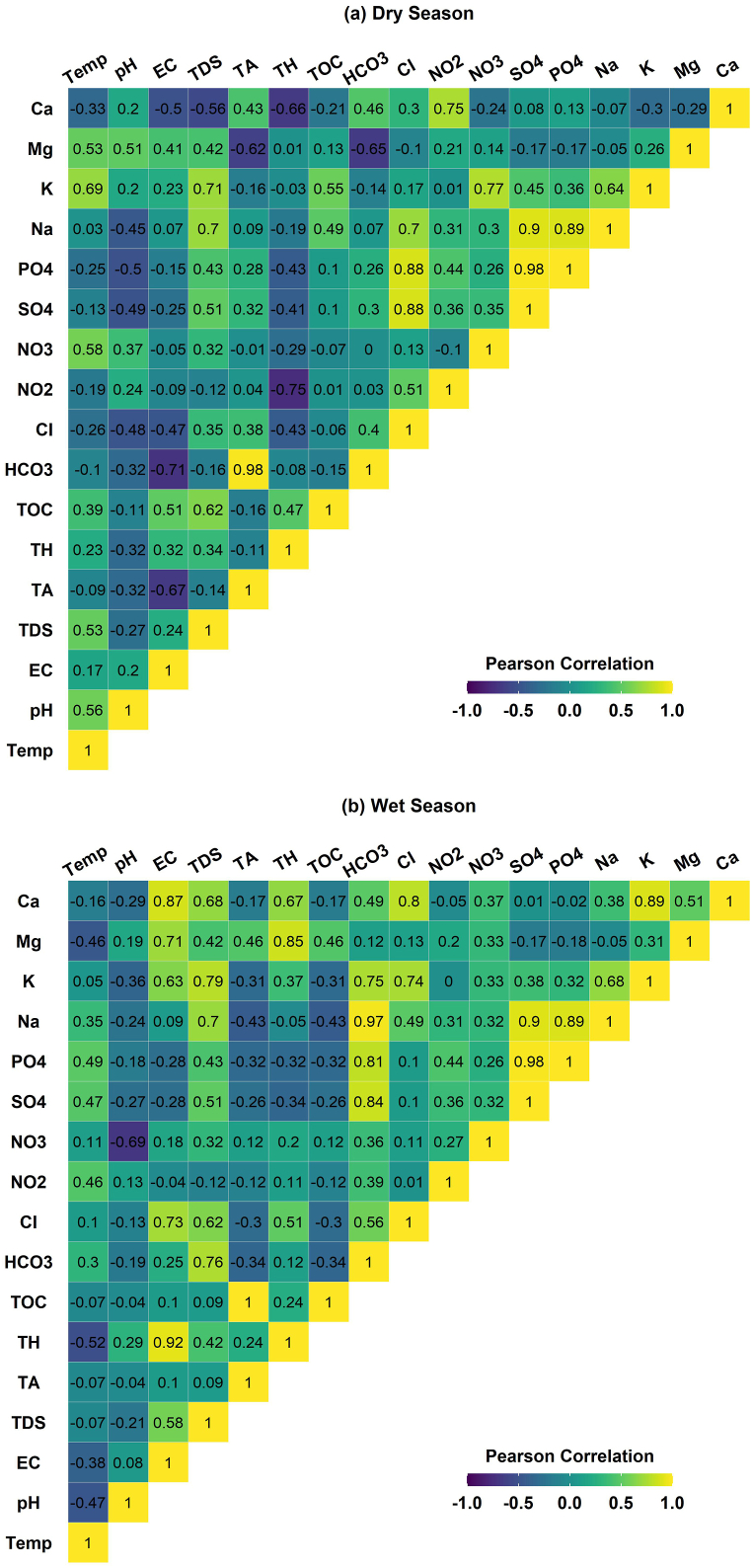
Correlation of among water quality indicators.

For the determination of the reliability of the PCA and CA results, the present study also performed Pearson correlation analysis. The correlation results are presented in Fig. 6. As shown in Fig. 6, significant positive associations were found among Cl^−^, PO_4_^3−^, SO_4_^2−^ and Na^+^ during the study period indicating a common origin of these ionic species. A strong positive correlation was found between TA and HCO_3_^−^ over the study period. Similar associations of both indicators were found in PCA and CA analysis that described in previous section. Like TA and HCO_3_^−^, similar associations were investigated for Ca and NO_2_^−^, both were showed a significant positive correlation between them during study period (Fig. 6). Correlation analysis. As discussed in earlier section above, EC, TOC, TH, K^+^ and Mg^2+^ were identified as a set of principal components and formed a cluster including these indicators. Similar associations were found among them in correlation analysis. In addition, Temp. And NO_3_ indicators shows a strong relationship between of them during both seasons (Fig. 6a; Fig. 6b). The results of the correlation revealed that the results of the PCA and CA were reliable to identify the sources of hydrogeochemical indicators.

### 3.4 Assessment of groundwater suitability

#### 3.4.1 Suitability of groundwater for drinking purposes

**Fig. 7:**
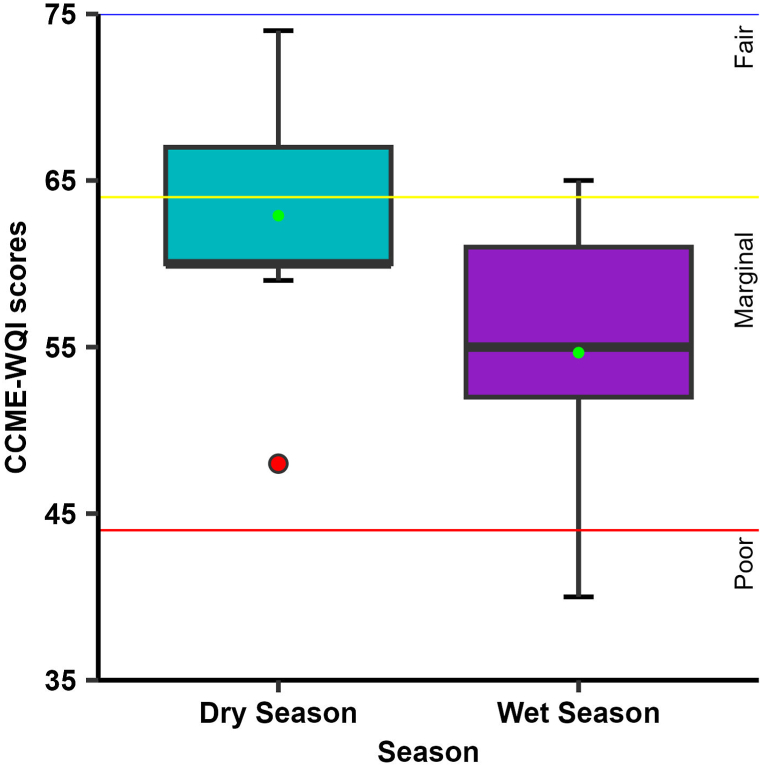
A statistical summary of the CCME-WQI score over the study period.

For evaluating the groundwater quality for drinking purposes, the CCME-WQI model was utilized since it is widely applied due to its ease of application and it provides flexibility in choosing the water quality indicators to be included in the model [8]. The CCME-WQI model was computed using the [60] standard limits as reference values. The statistical summary of the CCME-WQI score across the sampling locations during both the dry and wet seasons is presented in Fig. 7. Details of the CCME-WQI results can be found in Table S4. The overall status of water quality in the study area varied between “poor” to “marginal” whereas, most wells water quality were found “marginal” category (Table S4). Fig. 7 shows the water quality status of groundwater. Relatively, higher index scores were calculated over the dry season (ranged between 48.0 and 74.0) while it was lower in the wet season (ranged between 40.0 and 65.0 (Fig. 7).

**Fig. 8:**
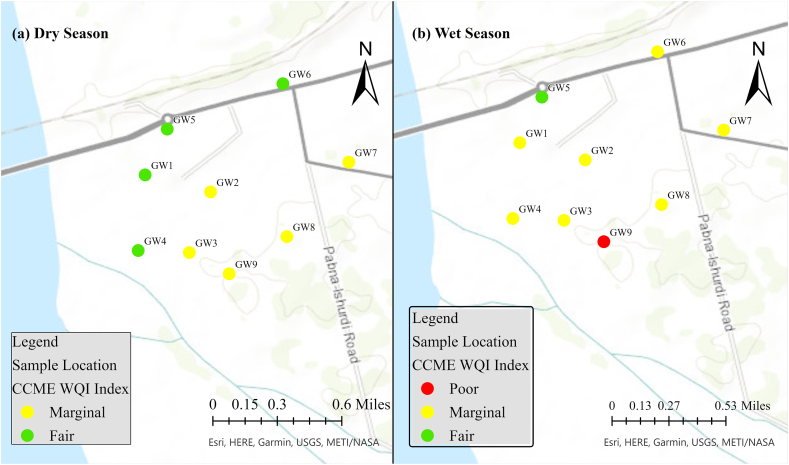
Groundwater quality status in the RNPP area.

According to the CCME WQI scores, during the dry season, 56.0% (5) of the sampling sites were classified as “marginal,” while 44.0% (4) of the sampling sites exhibited water quality that fell within the “fair” category (Fig. 8a). In contrast to the dry season, it was observed that the quality of water deteriorated during the wet season. The majority of the sampling sites indicated water quality falling under the “marginal” category, with the exception of GW5, which was categorized as “fair,” and GW9, which was categorized as “poor” (as depicted in Fig. 8b). Throughout the study period, it was observed that the water quality status for GW2, GW3, GW5, GW7, and GW8 remained consistent, with a marginal classification during both seasons, as depicted in Fig. 8.

Nonetheless, the findings of the CCME index suggest that the quality of groundwater was unsuitable for drinking purposes over the study period. The results of this investigation are consistent with prior research that has been recorded on the groundwater within the relevant area of study [4].

#### 3.4.2 Evaluation of groundwater for irrigation

**Fig. 9:**
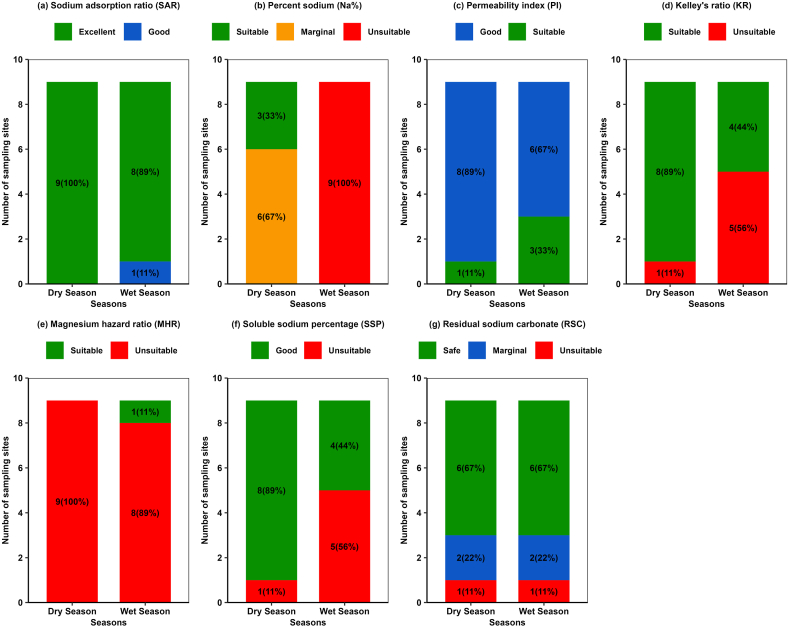
Irrigation water quality indices scores for groundwater within the sampling sites.

The initial evaluation was done by undertaking water quality indicators such as EC, TDS and TH. Values of EC and TDS were found within the good to permissible category throughout the study period while TH values indicated hard water at sampling site GW5, GW6 and GW7 during both season and at sampling site GW8 and GW9 during the dry season (Table 2, Table 3). Furthermore, in order to assess groundwater quality for irrigation purposes, the present study was utilized various indices approaches, including the Sodium adsorption ratio (SAR), Percent sodium (Na%), Permeability index (PI), Kelley's ratio (KR), Magnesium hazard ratio (MHR) and Soluble sodium percentage (SSP) that are widely used in evaluating groundwater in terms of irrigation purposes. Fig. 9 presents a statistical summary of various irrigation indices over the study period. Details of the classification schemes for each of the indices are provided in Table 2. The results of various indices are discussed below.

(i) SAR: Fig. 9a provides the results obtained from the SAR analysis. It is apparent from the figure that almost 100% and 89.0% samples of the groundwater were found to excellent for irrigations works during both seasons, respectively. This finding is consistent with that of [50] who reported around 90% groundwater samples from the similar region of interest were found in excellent in terms of irrigation purposes.
(ii) Percent of sodium: During the dry season, 67.0% (6) samples were ranked “marginally suitable” while 33.0% (3) samples were found as “suitable category” (Fig. 9b). Compared to dry season, higher index scores found for the wet season whereas all samples were ranked as “unsuitable” for irrigation activities (Fig. 9b). Unsuitable %Na score during wet period might be attributed from the enrichment of cations through runoff from arable lands [109].
(iii) PI: The PI is another important index in assessing the irrigation suitability as the soil permeability is impacted by the long-term association with irrigation water, influenced by sodium, calcium, magnesium, and bicarbonate contents of the soil [72]. Based on PI results, water classification in the study area, all groundwater samples fall within the “good” to “suitable” class for irrigation purposes (Fig. 9c).
(iv) KR: The KR index widely used to determine the impact of sodium content over calcium and magnesium [73]. Higher KR values (>1) indicate to the presence of excessive amount of dissolved Na in the groundwater, which could lead to undesirable effects in soil properties [18]. Therefore, groundwater with a KR < 1 is considered fit for irrigation purposes, while KR > 1 appraise unfit water for irrigation. In this study, Only 11.0% (1) of samples scored KR value greater than 1 during the dry season while in the wet season it increased to 56.0% (5) of samples (Fig. 9d). This substantial alteration during the wet season might be influenced by the rainfall associated runoffs containing agrochemicals [110].
(v) MHR: Generally, the Ca and Mg remain in balance in natural waters and both of these alkaline earth metals are considered as the essential nutrients for plants [111]. However, elevated levels of Mg and Ca in irrigation water can increase the soil pH with a consequential decrease in phosphorous availability and thus decrease the crop's yield [112]. The MHR index compares the Mg level in association with Ca in irrigation waters and indicate Mg hazard. Surprisingly, almost 100% samples were found unsuitable during the dry season, and it reduced to 89.0% samples during the wet season (Fig. 9e). Several studies have reported that the elevated concentration of Mg^2+^ could yield the alkaline agricultural soil that might hamper the soil productivity [113]. Compared to wet season, higher levels of Mg^2+^ was found during the dry season that might have resulted in higher MHR values throughout the sampling sites (Table 3).
(vi) SSP: Around 89.0% of samples were below 50.0 for SSP that pointed out majority of the sampling site's water was suitable for irrigation in the dry season and this ratio changed during the wet season when around 56.0% of samples shifted to not suitable for irrigation (Fig. 9f).
(vii) RSC: The RSC was applied to determine the impacts of CO_3_^2−^/HCO_3_^−^ and Ca^2+^/Mg^2+^ on the groundwater used for agricultural purposes. The computed RSC values indicated majority of the groundwater samples were found safe (67.0%) for agricultural works whereas 22.0% and 11.0% samples were identified as marginal and unsuitable for the mentioned purposes throughout the study period (Fig. 9g). However, most recent study by Ref. [50] in the similar study location found around 80.0% samples in the marginal category that implies the critical condition of the groundwater for irrigation activities.

**Fig. 10:**
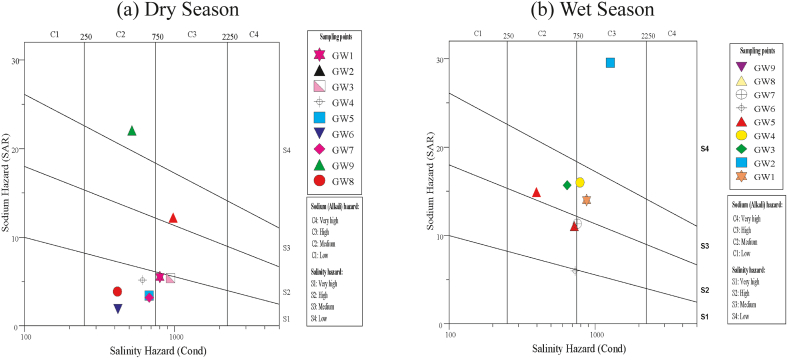
Wilcox diagram shows suitability water for irrigation purpose.

The present study utilized the US salinity diagram to assess the risk of sodium alkalinity in groundwater, which has been visualized through Fig. 10. As shown in Fig. 10a, most water samples, about 55.0% (5) samples in the dry season was under the C2S1 category while 22.0% (2) samples were under the C3S1 category supporting the earlier finding in the similar study area (52). In the wet season, the dominant categories were C2S2, C2S3 and C3S3 with having 22.2% samples in each of the category stating combined moderate to high salinity hazard (Fig. 10b).

### 3.5 Implications and limitations of the study

For the purposes of the assessment of the hydro-geochemical status of the groundwater resources around the RNPP area of Bangladesh, present study utilized the dataset prior to the RNPP construction period (2014–2015). Additionally, the current study appraised the suitability of groundwater resources for drinking and irrigation purposes during the above-mentioned period. In order to achieve the study aim, present study included 17 water quality indicators from nine sampling sites within and around the RNPP area. Present study applied a range of tools for assessing the hydro-geochemical features of groundwater and its suitability for irrigation and drinking purposes. In terms of hydro-geochemical assessment, the Piper trilinear diagram analysis revealed the overall dominance of alkaline earth and weak acids on the groundwater hydro-geochemistry that was also reported prior to this study [51]. Moreover, recent studies have confirmed the stable mechanism of alkaline earth-weak acids for controlling the hydro-geochemistry of the study domain's groundwater resources [50]. For the purposes of apprising the suitability of groundwater for drinking purposes, this study applied the CCME-WQI model. The CCMW-WQI model demonstrated that the groundwater ranked between “fair” to “marginal” during the dry season whereas “marginal” to “poor” status of groundwater was found during the season. In accordance with the present results of CCME-WQI model, previous studies have demonstrated similar status of groundwater quality for human consumption [4]. The unsuitable drinking water during the wet season might be attributed from the enrichment of contaminants due to rainfall-induced runoff. Finally, present study employed several irrigation indices as outlines in Table 2. Following the trend of drinking water, suitable groundwater for irrigation activities were also found during the dry season. Thus based on the findings of this, this research accepted the null hypothesis. From a theoretical perspective, although recent literature is in line with present outcome, continued monitoring of groundwater resources are required for sustainable water resources management, considering the imminent operation of RNPP. In this regard, baseline data would be very efficient for current and future monitoring efforts.

Turning to the practical standpoint of this research work, it is worth mentioning that the sustainable management of water resources in often challenging due to financial compliance, institutional framework, requirement of sophisticated analytical procedure and establishment, lack of skilled human resources [ [31,[114], [115], [116], [117], [118], [119]]]. Developed countries in the world mitigate these challenges through regional management tools (e.g., Water Framework Directive for European countries) but it remains as a tremendous challenge for developing countries like Bangladesh. Although, the recent water environment monitoring system of Bangladesh is advancing with a state-of-art monitoring practices, this system was not in service few years back. Consequently, it was not possible to assess prior environmental condition for several extreme hotspot areas within Bangladesh including areas adjacent to RNPP. Since groundwater is a vital resource for achieving SDG 6, its safeguarding requires a continuous set of data for signalling any changes in groundwater quality. In this regard, findings from this study provided the status of groundwater resources prior to the construction and operation of RNPP project that would serve as a baseline information for strategic planners for determining the impact of RNPP on the groundwater resources.

Although the utilization of 2014–2015 dataset constitutes the major limitation of this study, the practical implication of this research as stated above would have a pivotal role for the protection of groundwater resources adjacent to RNPP area. Looking ahead, future research should focus on the heavy metals contamination in groundwater as it is substantially interlinked with human health. In addition to that, the expansion of the spatial coverage would enhance the credibility of forthcoming research works. Furthermore, baseline data and continued monitoring efforts are required in order to develop a data repository for groundwater resources around the RNPP area.

## 4 Conclusion

The purpose of the current study was to assess the groundwater hydro-geochemistry and its suitability for human consumption and irrigation activities for the year 2014–2015 in the adjacent areas of RNPP. For achieving the study goal, this study utilized several hydrogeological tools (e.g., Piper diagram analysis) and statistical analysis (e.g., multivariate statistics) for assessing the hydro-geochemistry of groundwater samples. Moreover, CCME-WQI model and irrigation indices were employed for appraising groundwater's suitability in terms of drinking and irrigations purposes, respectively. Based on the results, the findings of this study are summarized as follows. Firstly, majority of the water quality indicators were found within the permissible limit of WHO and ECR apart from the TA, PO_4_^3−^ and K^+^ over the study period. The second major finding indicated that, Ca^2+^-Mg^2+^-HCO_3_^-^ substantially influenced the groundwater hydro-geochemistry especially during the dry season. However, the dominance of alkalis (Na^+^ and K^+^) and strong acids (Cl^−^ and SO_4_^2−^) prevailed during the wet season due to enrichment of TDS in the aquifer. Thirdly, the multivariate analysis pointed out the co-occurrence of anions such as Cl^−^, SO_4_^2−^, PO_4_^3−^ and NO_2_^−^ and cation Na^+^ and Ca^2+^ from anthropogenic input. Finally, both CCME-WQI model and irrigations indices implied the suitability of groundwater during the dry season. The unsuitable status of groundwater quality during the wet season could be due to the enrichment of contaminants in the aquifer from rainfall-induced runoffs.

The major limitation of this research is that the groundwater quality data was retrieved in 2014–2015. Despite its limitations, the study contributes to the understanding of groundwater chemistry for a critically hotspot area like RNPP. Moreover, the findings of this study could be useful as background information for managing groundwater quality and improving the existing monitoring program. Therefore, additional research should be conducted to continue enriching the baseline database, which could be useful in determining the impact of RNPP on groundwater and improving future research.

## Author contribution statement

Md Galal Uddin: Conceived and designed the experiments, Performed the experiments, Analyzed and interpreted the data, Contributed reagents, materials, analysis tools or data, Wrote the paper. Mir Talas Mahammad Diganta: Conceived and designed the experiments, Analyzed and interpreted the data, Contributed reagents, materials, analysis tools or data, Wrote the paper. Abdul Majed Sajib: Analyzed and interpreted the data. Md. Abu Hasan: Performed the experiments, Contributed reagents, materials, analysis tools or data, Wrote the paper. Md. Moniruzzaman: Contributed reagents, materials, analysis tools or data, Wrote the paper. Azizur Rahaman: Analyzed and interpreted the data, Wrote the paper. Agnieszka I. Olbert: Analyzed and interpreted the data, Wrote the paper. Md. Moniruzzaman: Analyzed and interpreted the data, Wrote the paper.

## Data availability statement

No data was used for the research described in the article.

## Declaration of competing interest

The authors declare that they have no known competing financial interests or personal relationships that could have appeared to influence the work reported in this paper.
