# Supplementary material for: Assessment of hydrogeochemistry in groundwater using water quality index model and indices approaches
Source: Heliyon. 2023 Sep 9;9(9):e19668. doi: 10.1016/j.heliyon.2023.e19668 (PMC10558938; doi:10.1016/j.heliyon.2023.e19668)
Supplement: Multimedia component 1 [file mmc1.docx]

**Supplementary material:**

**Assessment of hydrogeochemistry in groundwater using water quality index model and indices approaches**

**Md Galal Uddin^a,b,c,d,e*^, Mir Talas Mahammad Diganta^a,b,c,d^, Abdul Majed Sajib^a,b,c,d^, Md. Abu Hasan^f^, Md. Moniruzzaman^f^, Azizur Rahman^g,h^, Agnieszka I. Olbert^a,b,c,d^, Md. Moniruzzaman^e^**

^a^ Civil Engineering, School of Engineering, College of Science and Engineering, University of Galway, Ireland

^b^ Ryan Institute, University of Galway, Ireland

^c^ MaREI Research Centre, University of Galway, Ireland

^d^ Eco-HydroInformatics Research Group (EHIRG), Civil Engineering, University of Galway, Ireland

^e^ Department of Geography and Environment, Jagannath University, Dhaka, Bangladesh

^f^ Bangladesh Reference Institution for Chemical Measurements (BRiCM), Dr. Qudrat-e- Khuda Road, Dhanmondi, Dhaka 1205, Bangladesh

^g^ School of Computing, Mathematics and Engineering, Charles Sturt University, Wagga Wagga, Australia

^h^ The Gulbali Institute of Agriculture, Water and Environment, Charles Sturt University, Wagga Wagga, Australia

^*^ Corresponding author: Md Galal Uddin, Post-doctoral Researcher, Civil Engineering, School of Engineering, College of Science and Engineering, University of Galway, Ireland. E-mail address: [mdgalal.uddin@universityofgalway.ie](mailto:mdgalal.uddin@universityofgalway.ie)


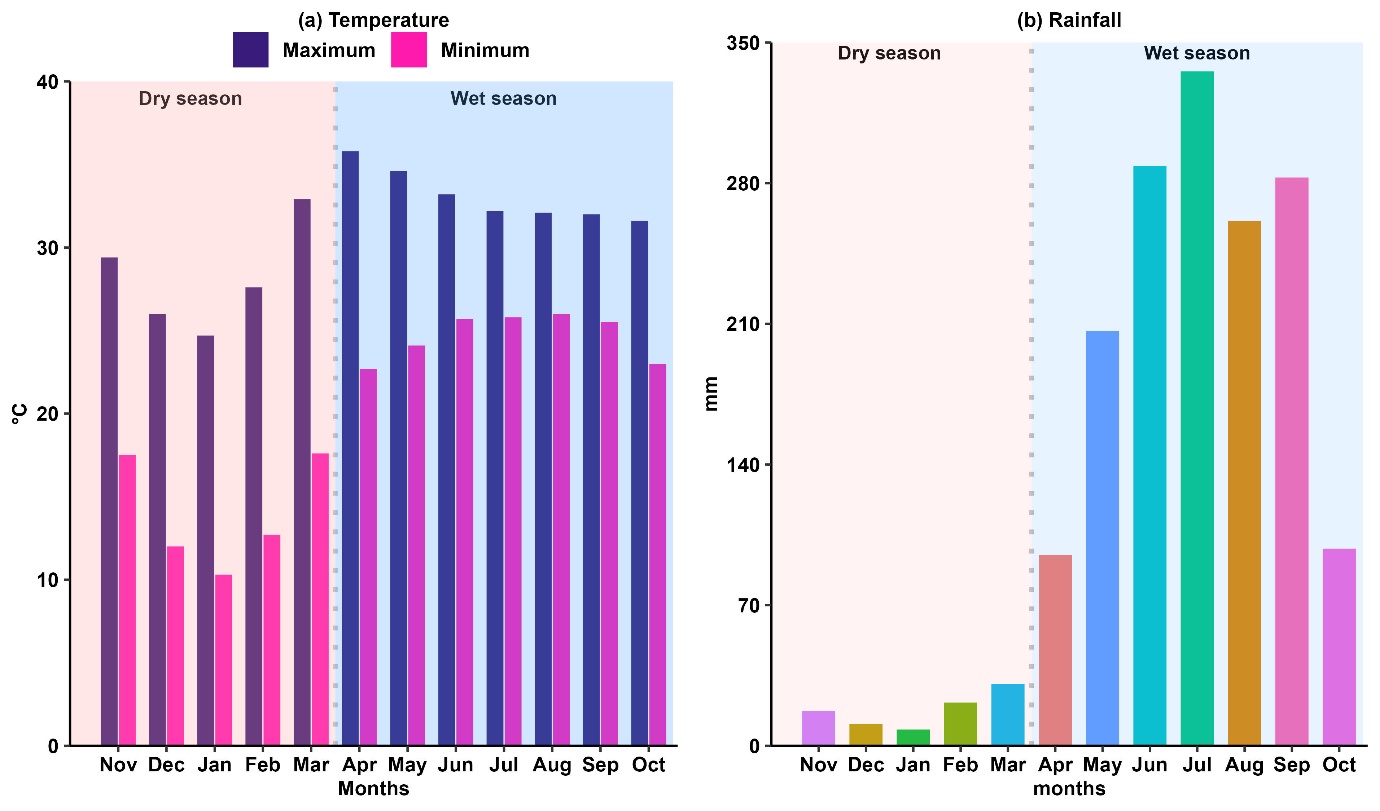


Fig. S1. Meteorological attributes of the study domain.

Table S1. CCME WQI index categorization scheme [1].

| Rank | WQI value | Description |
| --- | --- | --- |
| Excellent | 95.0-100 | Water quality is protected with a virtual absence of threat or impairment; conditions very close to natural or pristine levels |
| Very Good | 89.0-94.0 | Water quality is protected with a slight presence of threat or impairment conditions close to natural or pristine levels |
| Good | 80.0-88.0 | Water quality is protected with only a minor degree of threat or impairment, conditions rarely depart from natural or desirable levels |
| Fair | 65.0-79.0 | Water quality is usually protected but occasionally threatened or impaired; conditions sometimes depart from natural or desirable levels |
| Marginal | 45.0-64.0 | Water quality is frequently threatened or impaired; conditions often depart from natural or desirable levels |
| Poor | 0.00-44.0 | Water quality is almost always threatened or impaired; conditions usually depart from natural or desirable levels |

Table S2. Comparison of studied groundwater quality indicators with other studies from Bangladesh.

| Study area | Ishwardi | | | | | Rajshahi | Sylhet | Dhaka | Comilla | Jashore | Kushtia | Tangail |
| --- | --- | --- | --- | --- | --- | --- | --- | --- | --- | --- | --- | --- |
| Reference | This study | Hossain et al. (2010) [2] | Uddin et al. (2017) [3] | Choudhury et al. (2022) [4] | Rahman et al. (2022) [5] | Mostafa et al. (2017) [6] | Ahmed et al. (2019) [7] | Sharmin et al. (2020) [8] | Saha and Rahman (2020) [9] | Kabir et al. (2021) [10] | Islam and Mostafa (2022) [11] | Al-Asad et al. (2023) [12] |
| Temp. | 24.9 | - | 26.3 | - | - | 27.4 | 25.6 | - | - | - | - | - |
| pH | 7.67 | - | 6.55 | - | 7.66 | 6.91 | 5.69 | 6.54 | 7.10 | 7.94 | 7.42 | 6.67 |
| EC | 710 | - | 793 | - | 277 | 445 | 292 | 459 | 242 | 868 | 813 | 471 |
| TDS | 261 | - | - | - | 167 | 297 | - | 321 | 165 | 567 | 507 | 228 |
| TA | 129 | - | 327 | - | - | - | - | - | - | - | 183 | - |
| TH | 226 | - | - | - | - | 304 | - | 153 | - | 287 | 383 | - |
| TOC | 2.97 | - | - | - | - | - | - | - | - | - | - | - |
| HCO_3_^-^ | 64.0 | 392 | - | - | 23.8 | 245 | 123 | 185 | 291 | 405 | 433 | 207 |
| CI^-^ | 43.6 | 75.3 | 17.1 | - | 15.5 | 55.3 | - | 21.2 | 98.8 | 43.5 | 29.1 | 21.8 |
| PO_4_^3-^ | 15.6 | 5.94 | - | - | - | - | - | 0.060 | 3.95 | 0.010 | 0.949 | - |
| SO_4_^2-^ | 11.4 | 3.07 | 4.79 | 8.13 | 12.1 | 38.7 | 1.4 | 11.2 | 4.70 | 0.900 | 15.8 | 15.6 |
| NO_2_^-^ | 0.480 | - | 3.33 | - | - | - | - | - | - | 5.04 | - | - |
| NO_3_^-^ | 8.41 | 1.70 | 31.7 | 8.52 | - | 1.60 | 5.71 | - | 0.550 | 10.6 | 3.93 | 6.50 |
| Na^+^ | 60.5 | 27.3 | 15.1 | 7.35 | 10.7 | - | 39.4 | 28.6 | 77.5 | 68.3 | 12.8 | 21.2 |
| K^+^ | 75.8 | 7.13 | 3.13 | 3.43 | 1.48 | - | 2.33 | 2.13 | 7.80 | 1.38 | 1.14 | 5.45 |
| Ca^+^ | 16.0 | 67.6 | - | 78.9 | 14.0 | - | 7.62 | 37.8 | 31.0 | 67.7 | 105 | 46.5 |
| Mg^+^ | 24.5 | 42.1 | 25.3 | 22.8 | 4.71 | - | 4.83 | 12.8 | 33.7 | 27.4 | 30.8 | 12.8 |

Table S3. Temporal variation of water quality indicators over the study period (Significance level p < 0.05).

| WQ indicators | df | t | p-value |
| --- | --- | --- | --- |
| Temp. | 10.4 | 9.98 | < 0.050 |
| pH | 8.66 | 2.23 | 0.053 |
| EC | 15.6 | 0.730 | 0.476 |
| TDS | 8.35 | 4.76 | < 0.050 |
| TA | 14.2 | -0.774 | 0.451 |
| TH | 8.27 | 5.25 | < 0.050 |
| TOC | 14.6 | 0.613 | 0.548 |
| HCO_3_^-^ | 14.2 | -0.765 | 0.456 |
| Cl^-^ | 10.1 | 5.36 | < 0.050 |
| PO_4_^3-^ | 13.1 | 0.675 | 0.511 |
| SO_4_^2-^ | 10.7 | 0.601 | 0.559 |
| NO_2_^-^ | 9.75 | 6.11 | < 0.050 |
| NO_3_^-^ | 11.7 | 2.05 | 0.063 |
| Na^+^ | 10.1 | 1.94 | 0.079 |
| K^+^ | 8.38 | 7.07 | < 0.050 |
| Ca^2+^ | 15.4 | 1.34 | 0.197 |
| Mg^2+^ | 12.6 | -5.68 | < 0.050 |

Table S4. Water quality status of groundwater adjacent to the Rooppur nuclear power plant in Ishwardi, Bangladesh.

| Sampling sites | Dry Season | | Wet Season | |
| --- | --- | --- | --- | --- |
|  | CWQI | Rank | CWQI | Rank |
| GW1 | 65.0 | Fair | 53.0 | Marginal |
| GW2 | 59.0 | Marginal | 52.0 | Marginal |
| GW3 | 60.0 | Marginal | 55.0 | Marginal |
| GW4 | 67.0 | Fair | 61.0 | Marginal |
| GW5 | 73.0 | Fair | 65.0 | Fair |
| GW6 | 74.0 | Fair | 63.0 | Marginal |
| GW7 | 60.0 | Marginal | 47.0 | Marginal |
| GW8 | 60.0 | Marginal | 55.0 | Marginal |
| GW9 | 48.0 | Marginal | 40.0 | Poor |

References

[1] Uddin, M.G., Nash, S., Olbert, A.I., 2021. A review of water quality index models and their use for assessing surface water quality. Ecol. Indic. 122, 107218. <https://doi.org/10.1016/j.ecolind.2020.107218>

[2] Hossain, G., Howladar, M.F., Nessa, L., Ahmed, S.S., Quamruzzaman, C., 2010. Hydrochemistry and Classification of Groundwater Resources of Ishwardi Municipal Area, Pabna District, Bangladesh. Geotech. Geol. Eng. 28, 671–679. <https://doi.org/10.1007/s10706-010-9326-4>

[3] Uddin, M.G., Moniruzzaman, M., Khan, M., 2017. Evaluation of Groundwater Quality Using CCME Water Quality Index in the Rooppur Nuclear Power Plant Area, Ishwardi, Pabna, Bangladesh. Am. J. Environ. Prot. 5, 33–43. <https://doi.org/10.12691/env-5-2-2>

[4] Choudhury, T.R., Ferdous, J., Haque, M.M., Rahman, M.M., Quraishi, S.B., Rahman, M.S., 2022. Assessment of heavy metals and radionuclides in groundwater and associated human health risk appraisal in the vicinity of Rooppur nuclear power plant, Bangladesh. J. Contam. Hydrol. 251, 104072. <https://doi.org/10.1016/j.jconhyd.2022.104072>

[5] Rahman, M.S., Parvez, S., Begum, B.A., Quraishi, S.B., Choudhury, T.R., Fatema, K.J., Hosen, M.M., Bodrud-Doza, M., Rahman, L.S., Sattar, M.A., 2022. Chemometric appraisal of water quality for domestic and agricultural purposes: a case study from establishing Rooppur Nuclear Power Plant (NPP) area, Pabna District, Bangladesh. Environ. Sci. Pollut. Res. 29, 56620–56641. <https://doi.org/10.1007/s11356-022-19308-6>

[6] Mostafa, M.G., Uddin, S.M.H., Haque, A.B.M.H., 2017. Assessment of hydro-geochemistry and groundwater quality of Rajshahi City in Bangladesh. Appl. Water Sci. 7, 4663–4671. <https://doi.org/10.1007/s13201-017-0629-y>

[7] Ahmed, N., Bodrud-Doza, M., Islam, S.M.D.U., Choudhry, M.A., Muhib, M.I., Zahid, A., Hossain, S., Moniruzzaman, M., Deb, N., Bhuiyan, M.A.Q., 2019. Hydrogeochemical evaluation and statistical analysis of groundwater of Sylhet, north-eastern Bangladesh. Acta Geochim. 38, 440–455. https://doi.org/10.1007/s11631-018-0303-6

[8] Sharmin, S., Mia, J., Miah, M.S., Zakir, H.M., 2020. Hydrogeochemistry and heavy metal contamination in groundwaters of Dhaka metropolitan city, Bangladesh: Assessment of human health impact. HydroResearch 3, 106–117. https://doi.org/10.1016/j.hydres.2020.10.003

[9] Saha, N., Rahman, M.S., 2020. Groundwater hydrogeochemistry and probabilistic health risk assessment through exposure to arsenic-contaminated groundwater of Meghna floodplain, central-east Bangladesh. Ecotoxicol. Environ. Saf. 206, 111349. https://doi.org/10.1016/j.ecoenv.2020.111349

[10] Kabir, M.M., Hossain, N., Islam, A.R.M.T., Akter, S., Fatema, K.J., Hilary, L.N., Hasanuzzaman, M., Didar-ul-Alam, M., Choudhury, T.R., 2021. Characterization of groundwater hydrogeochemistry, quality, and associated health hazards to the residents of southwestern Bangladesh. Environ. Sci. Pollut. Res. 28, 68745–68761. https://doi.org/10.1007/s11356-021-15152-2

[11] Islam, M.S., Mostafa, M.G., 2022. Suitability of water quality index methods for assessing groundwater quality in the Ganges River basin area. H2Open J. 5, 198–220. https://doi.org/10.2166/h2oj.2022.145

[12] Al- Asad, H., Moniruzzaman, M., Sarker, A.K., Quaiyum Bhuiyan, M.A., Ahsan, M.A., 2023. Hydrogeochemical evaluation, groundwater contamination and associated health risk in southern Tangail, Bangladesh. Chemosphere 332, 138806. https://doi.org/10.1016/j.chemosphere.2023.138806
